# An experimental study on breast lesion detection and classification from ultrasound images using deep learning architectures

**DOI:** 10.1186/s12880-019-0349-x

**Published:** 2019-07-01

**Authors:** Zhantao Cao, Lixin Duan, Guowu Yang, Ting Yue, Qin Chen

**Affiliations:** 1 The Big Data Research Center, University of Electronic Science and Technology of China, No.2006, Xiyuan Ave, West Hi-Tech Zone, Chengdu, 611731 China; 2 School of Medicine, University of Electronic Science and Technology of China, No.2006, Xiyuan Ave, West Hi-Tech Zone, Chengdu, 611731 China; 3 Sichuan Academy of Medical Sciences & Sichuan Provincial People’s Hospital, University of Electronic Science and Technology of China, No.32 West Second Section First Ring Road, Chengdu, 610072 China

**Keywords:** Deep Learning, Computer-Aided Diagnosis, Breast Lesion Detection, Breast Lesion classification

## Abstract

**Background:**

Computer-aided diagnosis (CAD) in the medical field has received more and more attention in recent years. One important CAD application is to detect and classify breast lesions in ultrasound images. Traditionally, the process of CAD for breast lesions classification is mainly composed of two separated steps: i) locate the lesion region of interests (ROI); ii) classify the located region of interests (ROI) to see if they are benign or not. However, due to the complex structure of breast and the existence of noise in the ultrasound images, traditional handcrafted feature based methods usually can not achieve satisfactory result.

**Methods:**

With the recent advance of deep learning, the performance of object detection and classification has been boosted to a great extent. In this paper, we aim to systematically evaluate the performance of several existing state-of-the-art object detection and classification methods for breast lesions CAD. To achieve that, we have collected a new dataset consisting of 579 benign and 464 malignant lesion cases with the corresponding ultrasound images manually annotated by experienced clinicians. We evaluate different deep learning architectures and conduct comprehensive experiments on our newly collected dataset.

**Results:**

For the lesion regions detecting task, Single Shot MultiBox Detector with the input size as 300×300 (SSD300) achieves the best performance in terms of average precision rate (APR), average recall rate (ARR) and F_1_ score. For the classification task, DenseNet is more suitable for our problems.

**Conclusions:**

Our experiments reveal that better and more efficient detection and convolutional neural network (CNN) frameworks is one important factor for better performance of detecting and classification task of the breast lesion. Another significant factor for improving the performance of detecting and classification task, which is transfer learning from the large-scale annotated ImageNet to classify breast lesion.

## Background

Breast cancer is the second leading cause of female death. Early diagnosis is the key to breast cancer control, as it can reduce mortality dramatically (40% or more) [1]. Previously, mammography is the main modality for detecting breast cancer. However, mammography not only causes health risks for patients but also leads to unnecessary (65%-85%) biopsy operation due to low specificity [1]. As a much better option, ultrasound imaging can increase the overall cancer detection by 17% and reduce unnecessary biopsies by 40% [1].

Currently, ultrasound techniques for breast lesion detection rely on doctor’s experience, especially for the marks and classifications of breast lesions, the process is as follow: doctors use ultrasound instruments to find a good angle to make the lesions clearly shown on the screen, and then keep probe fixed for a long time using one hand, with another hand to mark and measure the lesion on the screen. It is a difficult task, because slight shaking of the hand which holds probe will cause a big impact on the quality of breast ultrasound images; and then, other doctors diagnose the ultrasound images, based on his experience, but it is usually hard to draw conclusion that lesions are benign or malignant, due to the complex structure of breasts and the existence of noise in the ultrasound images. Based on the above, automatically locating regions of interest (i.e., lesions) and classification (i.e, benign or malignant) is highly demanded breast lesion detection in ultrasound images.

Previous many researchers have analyzed the detection and classification of lesions in breast ultrasound images. We review the literature in the remainder of this section.

### Classification

In early machine learning, the mainstream machine learning methods were based on statistics, and they did not care about features. However, computer vision is the application of machine learning in the field of vision, which a good feature extraction method is crucial. Feature extraction is a process of dimension reduction, which reduce the number of resources needed for processing without losing important or relevant information, and facilitate the speed of learning and generalization steps in the machine learning process. There were a lot of manual feature extraction methods that can be divided into three categories [2, 3]: i) Interest point detection (such as Laplacian of Gaussian, Difference of Gaussian, Harris Corner Detection, Features From Accelerated Segment Test), ii) Dense features [4] (such as Scale Invariant Feature Transform [5], Histogram of Oriented Gradient [6], Local Binary Pattern [7, 8]), iii) Feature Combinations (such as Deformable Part-based Model [9, 10]).

Several previous methods discussed on how to automatically classify breast lesions. In [11], the authors built three M-dimensional feature sets and selected the features by principal component analysis and mutual information to classify 641 ultrasound images. In [12], the authors segmented the Breast Ultrasound images based on watershed transform and extracted 22 morphological features from segmented lesions, and selected the features based on mutual information and statistical tests to classify 641 ultrasound images. In [13], the authors proposed a computer-aided diagnosis method depending on the lesion’s shape type of ultrasound image. They used Zernike moments and invariant moment to extract feature, meanwhile, they used support vector machine and multilayer perceptron to classify 45 ultrasound images. In [14], The authors proposed a classified method by using texture analysis to extract features, and perceptron classification method was used to classify 57 ultrasound images. In [15], the authors classed the primary and secondary occurring of benign and malignant cases. they extracted Laws’ mask texture features from the ultrasound images and used support vector machine as a classifier to distinguish 172 ultrasound images of the breast lesions.

### Detection

In the past, researchers usually studied hand-crafted features within the traditional detection framework. For example, Dalal et al. [6] used support vector machine with the Histogram of Oriented Gradients features for the pedestrian detection task. Felzenszwalb et al. [9, 10] proposed a Deformable Part-based Model using latent support vector machine, which achieved the best performance in the 2006 Pattern Analysis, Statistical Modelling and Computational Learning person detection challenge. In [16], the authors used the dictionary learning method to obtain a sparse expression of an image, which was called Histograms of Sparse Codes. Histograms of Sparse Codes was used to replace Histogram of Oriented Gradients for classifier training and target detection. Although the performance has been considerably improved, the detection speed is quite slow. In [17], the author proposed an object detector based on co-occurrence features, which was three kinds of local co-occurrence features constructed by the traditional Harris Corner Detection, Local Binary Pattern, and Histogram of Oriented Gradients respectively.

Several previous methods discussed on how to automatically locate ROI of breast lesions. In [18], A self-organizing map neural network was used for the detection of the breast lesion. The ROI can be extracted automatically by employing local textures and a local gray level co-occurrence matrix which is a joint probability density function of two positions. Compared with the basic texture feature, the gray level co-occurrence matrix can reflect the comprehensive information about the direction, the interval and the amplitude of the image. In [19], Shan et al. developed an automatic ROI generation method which consisted of two parts: automatic seed point selection and region growing. However, the method depends on textural features, and these features are not effective for breast ultrasound images when there exists a fat region close to the lesion area or contrast is low. In [20], a supervises learning method was proposed to categorize breast tissues into different classes by using a trained texture classifier, where background knowledge rules were used to select the final ROI for the tissues. However, due to the inflexibility of the introduced constraints in the proposed method, its robustness was reduced. In [21], the authors improved the method in [20] by proposing a fully automatic and adaptive ROI generation method with flexible constraints. In their work, the ROI seed can be generated with high accuracy, and can also well distinguish the datasets lesion regions from normal regions. However, as shown in the experiments, the recall is still unsatisfactory, that average recall rate was low that benign was 27.69%, malignant was 30.91%, the total was 29.29%.

Recently, deep learning techniques have attracted a lot of attention from researchers, because of the good data interpretability as well as the high discriminable power. Noticeably, deep convolutional neural network (CNN) have substantially improved the performance not only for general object detection [22–26], but also for image classification [27–32]. So far in the literature, people have employed CNN based methods to handle detection and classification tasks for medical images, such as mammograms [33]. To the best of our knowledge, there is little work that has comprehensively evaluated the performance of different CNN based detection and classification methods for lesions in breast ultrasound images.

## Methods

In this study, we analyze, explore and evaluate different object detection and classification methods based on CNN architectures for lesion detection and classification in breast ultrasound images, which is extended based on our MICCAI workshop paper [34]. Firstly, we introduce data collection; Secondly, we analyze various architectures of object detection based CNN that are applicable to the breast ultrasound images; Finally, we describe how to utilize CNN to classify breast lesions and CNN transfer learning from no-medical to breast ultrasound images.

### Data collection

**Fig. 1:**
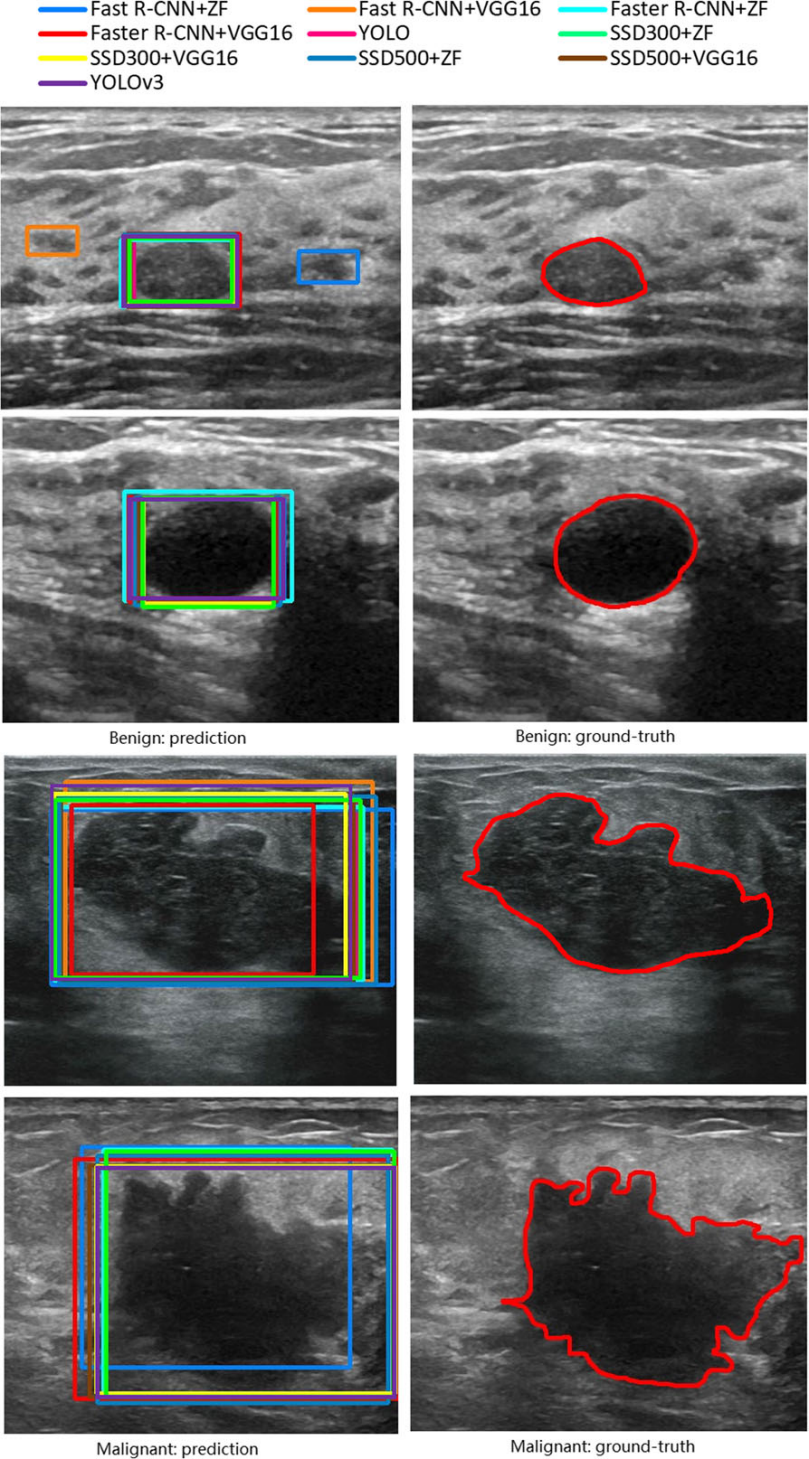
Ground-truth annotations and predicted bounding boxes of different methods, for four lesion cases from different patients

Collecting a well-defined dataset is key to the research on breast lesions detection/classification. For that, we have been collaborating with Sichuan Provincial People’s Hospital to have experienced clinicians annotate breast ultrasound images obtained from breast lesions patients. Specifically, the patients were told to get scanned by LOGIQ E9 (GE) and IU-Elite (PHILIPS) to generate those ultrasound images. Each ultrasound image was later reviewed and diagnosed by two or three clinicians. Based on the ratings obtained from the Breast Imaging-Reporting and Data System (BI-RADS) [35], each diagnosed image was then grouped into 7 categories indexed from 0 to 6, where 0 means more information is needed, 1 negative, 2 benign finding, 3 probably benign (less than 2% likelihood of cancer), 4 suspicious abnormality, 5 highly suggestive of malignancy, and 6 proven malignancy. According to [35], some medical specialists proposed to further partition the fourth category (suspicious abnormality) into three sub-category, i.e., 4A (low suspicion for malignancy), 4B (intermediate suspicion of malignancy) and 4C (moderate concern, but not obvious for malignancy). For that, by following the professional instructions from our clinicians, we divide our datasets into two classes: benign and malignant. The benign class is constructed by the images grouped into categories 2, 3 and 4A, while the malignant class consists of the images from categories 4B, 4C, 5 and 6. By working with the clinicians, we have collected 577 benign and 464 malignant cases from patients. Moreover, the lesion in each image has also been marked out by those experienced clinicians. Figure 1 show cases four ultrasound images containing either benign or malignant lesions. To the best of our knowledge, there is no such a publicly available ultrasound image datasets as ours for breast lesions.

### Training protocols of object detection

The remarkable progress of deep learning techniques, especially CNN, have largely promoted the research of visual object detection. Fast Region-based convolutional neural networks (R-CNN) [22], Faster R-CNN [23], You Only Look Once (YOLO) [24], YOLO version 3 (YOLOv3) [25], and Single Shot MultiBox Detector (SSD) [26] are existed state-of-the-art object detection methods. However, these CNN-based methods only focus on general object detection. In this paper, we apply them to detecting lesions in our newly collected breast ultrasound dataset. We also combine each CNN based detection method with different existing neural networks, e.g., Visual Geometry Group (VGG16) [29], ZFNet [28].

We use the result of method [21] as a baseline for the detection of breast lesions. All CNN are modified to evaluate these CNN architecture from ImageNet detection task to our dataset. Next, we will introduce the difference between these algorithms.

**Fast R-CNN** R-CNN [36] and Spatial Pyramid Pooling Net [37] using CNN to classify region proposals, and achieves excellent object detection accuracy. However, two major issues still exist: i) the training phase is a multi-stage pipeline; and ii) object detection is slow. To overcome these drawbacks, also inspired by Pyramid Pooling Net [37], Girshick et al. [22] improved R-CNN by proposing Fast R-CNN which adds an ROI pooling layer to the last convolution layer, the ROI pooling layer uses max pooling to convert the features inside any valid region of interest into a small feature map with a fixed spatial extent. Each feature is fed into a fully connected layers that finally branch into two output: one output produces softmax probability estimates and another output does bounding-box regression. In other words, performs classification and bounding box regression simultaneously.

**Faster R-CNN** Fast R-CNN, as selective search is used for region proposals, the detection time is not very fast. To avoid the standalone step to generate regions, Ren et al. [23] proposed to integrate a so-called Region Proposal Network (RPN) into Fast R-CNN, and RPN and fast R-CNN share large number of convolutional layers. In Faster R-CNN, an image as input fed into RPN and outputs a set of rectangular object proposals, each with an objectness score, which is fed into two sibling fully connected layers: an object category classification layer and a box regression layer, simultaneously regress objectness scores and region bounds at each location on a regular grid.

**YOLO** YOLO [24] employed a single convolutional neural network to predict the bounding boxes and class labels of detected regions. Since the YOLO limits the number of bounding boxes, it avoids repetitive detection of the same object and thus greatly improves the detection speed, making YOLO suitable for real-world applications. Due YOLO may fail to localize small objects, Redmon and Farhadi propose YOLO version 2 (YOLOv2) [38], an improved version of YOLO. YOLOv2 use a new classification model Darknet-19, and achieved state of the art on standard detection tasks. In [25], Redmon and Farhadi made a bunch of little design changes to YOLO, that present a faster and more accurate detecor than YOLOv2 which is called YOLOv3. YOLOv3 predicts bounding boxes with dimension priors and location. YOLOv3 use a much more powerful feature extractor network, which is a hybrid approach between the network used in YOLOv2, Darknet-19, and the newfangled residual network stuff. YOLOv3 is a fast and accurate detecor.

**SSD** In order to improve detection speed and accuracy, Liu et al. [26] proposed SSD, which only needs an input image and ground truth boxes for each object during training. For objects of different size, SSD adds several auxiliary convolutional feature layers which progressively decrease in size, and predicts detections at multi-scale. SSD uses shallower layers for detecting small objects. Furthermore, in a convolutional fashion, the SSD framework evaluate a small set of default boxes of different aspect ratios at each location in several feature maps with different scales. In order to efficiently to discretize the space of possible output box shapes allows different default box shapes in several feature maps. For each default box, SSD predicts both the shape offsets and the confidences for all object categories.

### Training protocols of classification

In this work, we mainly explore and evaluate different CNN architectures with different model training parameter values in classify breast lesions tasks. These CNN architectures learning from the labeled set, which has major advantages over more traditional approaches that use hand-crafted features. We also evaluate the transfer learning from no-medical datasets due to the lack of big data.

#### Convolutional neural network architectures

We mainly explore and evaluation AlexNet [27], ZFNet [28], VGG [29], ResNet [30], GoogLeNet [31], and DenseNet [32] with different model training parameter values in classify breast lesions tasks. These deep CNN architectures are described below.

##### AlexNet

The AlexNet [27] achieved significantly improved performance in ImageNet Large Scale Visual Recognition Competition (2012). AlexNet has five convolution layers, three fully-connected layers and has approximately 60 million free parameters.

##### ZFNet

The ZFNet architecture was published in [28], the author introduce a novel visualization technique that to reveal why CNN models perform so well. The architecture is based on AlexNet, which is an 8 layer convnet model which has five convolution layers, two fully-connected layers, and a softmax layer.

##### VGG

In VGG [29], the author main contribution is the evaluation of networks of increasing deep, which shows the depth to 16-19 weight layers that can significantly improve the performance. In this paper, we use 16 weight layers (VGG16) as default architecture.

##### GoogLeNet

GoogLeNet [31], the authors propose a new module called “Inception” which were based on the Hebbian theory and the intuition of multi-scale processing. The “Inception” layer consists of six convolution layers. The GooLeNet significantly increases the depth of the convolution network, more than 20 layers (two convolution layers and nine “Inception” module).

##### ResNet

In [30], the authors present a residual learning framework to solve the problem which difficult to train deeper CNN, and showing that these residual networks are easier to optimize. The framework explicitly reformulates the layers as learning residual functions. In our paper, we use 50-layers to evaluation and analysis our dataset.

##### DenseNet

DenseNet [32] connects each layer to every other layer in a feed-forward fashion. DenseNets have several advantages: Effectively solve the vanishing-gradient problem, reduce the number of parameters, feature reuse, and strengthen feature propagation. In this paper, the DenseNet-121 is our default DenseNet architecture for evaluation and analysis our dataset, and the growth rate is k = 32.

#### Training protocols

Previous many studies have analyzed lesion regions of interest which clinicians manual select ROI from full-size images (LROI) classification based traditional approaches. As we know, no existing work, which classifies breast lesions in ultrasound images have reached the performance requirements for a realistic clinical setting. In this paper, in order to system evaluation the influence of different architecture based CNN, but previously not care factors, we employ CNN to full-size image and LROI image classification. In order to accommodate the CNN architectures described above, all full-size images and LROI images were resized to 256×256 pixels and classified manually as either benign or malignant. We use the caffe framework to train all models, and we train for 2000 epoches which can observe the convergence.

Collecting and annotating large numbers of breast ultrasound images still poses significant challenges. Despite the disparity between natural images and breast ultrasound images, our hypothesis, CNN comprehensively trained on the large-scale well-annotated ImageNet may still be transferred to make medical image recognition tasks more effective. So, in this paper, we evaluate and analyze the influence of CNN models which not only learned from scratch, but also transfer learning from pre-trained models. When learned from scratch, all the random parameters of CNN models are initialized as follows: AlexNet, ZFNet, VGG with Gaussian random parameters; GoogLeNet with Xavier; ResNet and DenseNet with Microsoft Research Asia filler. For fine-tuned from pre-trained models, the last fully-connected layer is random initialized and freshly trained, in order to accommodate the new object categories in our task.

## Results

In this section, the experiments compare the performances of detection and classification methods based CNN on our dataset.

### Detection

In this paper, we compared the results of the different methods (the method in [21], Fast R-CNN, Faster R-CNN, YOLO, YOLOv3, SSD) on the locating lesion ROI in breast ultrasound images. For the deep architecture, we employ a medium-sized network VGG16 [29] and a small network ZFNet [28] for Fast R-CNN, Faster R-CNN, and SSD. We denote the detection architecture based on VGG16 as Fast+VGG16, Faster+VGG16, SSD300+VGG16, and SSD with the input size as 500×500 (SSD500)+VGG16; and denote the detection architecture based on ZFNet as Fast+ZFNet, Faster+ZFNet, SSD300+ZFNet, and SSD500+ZFNet. We denote the YOLO uses its original Darknet-53 model [24] as YOLO, and YOLOv3 uses its original Darknet53.conv.74 model [25] as YOLOv3.

For evaluation metric, we employ average precision rate (APR) and average recall rate (ARR) over all test images [21] as well as the F_1_ score for each method:

$$\begin{array}{@{}rcl@{}} \text{APR} &=& \frac{1}{N}\sum_{i=1}^{N}\frac{\left|R_{i}^{gt}\cap R_{i}^{pred}\right|}{\left|R_{i}^{pred}\right|}, \,\,\\ \text{ARR} &=& \frac{1}{N}\sum_{i=1}^{N}\frac{\left|R_{i}^{gt}\cap R_{i}^{pred}\right|}{\left|R_{i}^{gt}\right|}, \,\,\\ \mathrm{F}_{1} &=& \frac{2\times \text{APR} \times \text{ARR}}{\text{APR}+\text{ARR}}, \end{array} $$

where *N* is the number of images, $R_{i}^{gt}$ is the ground-truth lesion region, and $R_{i}^{pred}$ is the predicted bounding box. A higher APR shows the higher overlapped rate between the ROI and the true lesion region, while a higher ARR indicates that ROI generated by the proposed method could be subject to the removal of additional non-lesion regions.

In the experiments, we prepare our data as follows. For the benign class, 285 cases are randomly selected as the training set, 191 cases as the validation set and 103 cases as the test set. For the malignant class, we sample 230 cases as the training set, 154 cases as the validation set and 80 cases as the test set. In total (Benign + Malignant), we have 515 training cases, 345 validation cases, and 183 test cases. The comparison of these baselines is listed in Table 1, where the APRs, ARRs and F_1_ scores of different methods are compared on three settings, i.e., benign images only, malignant images only and both benign + malignant images. We can clearly observe that the CNN based methods perform much better than the method in [21]. In addition, in the CNN-based method, YOLO and SSD perform significantly better than Fast R-CNN and Faster R-CNN. Also, SSD300, in general, achieves good results than other CNN based methods, which shows SSD300 is more suitable for the lesion detection task in this work.

**Table 1:** APR, ARR and F_1_ scores of different methods under three settings

Method	Benign			Malignant			Benign+ Malignant		
	APR	ARR	F_1_	APR	ARR	F_1_	APR	ARR	F_1_
Auto ROI [21]	66.95	14.16	23.38	78.22	19.23	30.87	71.86	16.36	26.65
Fast+ZFNet	87.25	65.47	74.81	89.02	53.54	66.86	91.11	62.60	74.21
Fast+VGG16	90.17	66.39	76.47	71.00	40.83	51.84	88.70	61.97	72.96
Faster+ZFNet	93.14	66.25	77.43	86.37	46.83	60.73	92.42	62.23	74.38
Faster+VGG16	93.01	67.08	77.95	90.36	52.05	66.05	92.37	62.54	74.58
YOLO	95.59	68.85	80.05	96.46	57.73	72.23	96.81	65.83	78.37
YOLOv3	96.89	68.81	80.47	94.56	54.21	68.91	96.58	65.85	78.31
SSD300+ZFNet	**97.20**	**70.56**	**81.76**	96.44	54.91	69.97	**96.89**	**67.23**	**79.38**
SSD300+VGG16	96.03	69.76	80.82	**97.56**	**58.96**	**73.50**	96.42	66.70	78.85
SSD500+ZFNet	95.98	70.04	80.98	94.22	54.90	69.38	95.09	65.06	77.26
SSD500+VGG16	94.58	69.57	80.17	94.67	55.82	70.23	96.42	66.70	78.85

Note–Boldface data indicate the best results

We also plot the resultant bounding boxes predicted by different methods for four lesion cases in Fig. 1.

### Classification

In order to analyze the impact of learning for scratch and pretraining, we compared four different scenarios which were LROI with random initialization, LROI with transfer learning, full-size images with random initialization, and full-size images with transfer learning.

For evaluation metric, we employ accuracy rate (AR) for each method:

$$\begin{array}{@{}rcl@{}} \text{AR} = \frac{\left|B_{b}^{pre} + M_{m}^{pred}\right|}{\left|B_{b}^{pred} + M_{m}^{pred} + B_{m}^{pred} + M_{b}^{pred}\right|}, \end{array} $$

$B_{b}^{pre}$ is the number of images which the benign predict to benign, and $M_{m}^{pred}$ is the number of images which the malignant predict to malignant. $B_{m}^{pre}$ is the number of images which the benign predict to malignant, and $M_{b}^{pred}$ is the number of images which the malignant predict to benign. The matter that needs your attention is when a figure has more than one lesion, as long as there has a malignant lesion, this figure is malignant.

In the experiments, we prepare our data as follows. 476 cases in the benign class and 384 cases in the malignant class are randomly selected as the training set. And 103 cases benign class and 80 cases malignant class as the test set. In this experiment, we analyze and compare the performance of AlexNet, ZFNet, VGG16, GoogLeNet, ResNet, and DenseNet on our dataset. We conduct extensive empirical evaluation and compared four different scenarios which were described in above, and the result shown in Table 2. We can see the DenseNet achieves best results than other methods in all scenarios, which shows DenseNet is more suitable for our problems.

**Table 2:** Accuracy rates (AR) of different methods

Method	AlexNet	ZFNet	VGG16	GoogLeNet	ResNet	DenseNet
FULL-RI	56.6	56.7	56.9	69.6	75.0	**80.0**
FULL-FT	67.8	67.9	72.3	76.8	83.0	**85.0**
LROI-RI	60.0	66.3	56.7	68.8	75.0	**80.0**
LROI-FT	79.5	78.1	80.2	79.8	85.0	**87.5**

Note–Boldface data indicate the best results

## Discussion

### Detection

From Table 1, we can see YOLO and SSD perform significantly better than other methods. YOLO makes predictions based on each entire image so it implicitly encodes contextual information. There is no two-stage interception of ROI, so YOLO have fewer background errors. SSD add several auxiliary convolutional feature layers which progressively decrease in size, and predicts detections at multi-scale. SSD uses different layers for detecting the objects of different sizes. In the breast ultrasound image, there are many lesions of different sizes, and these advantages of SSD can also cover large and small lesion areas. It is worth noting that SSD300 is better than SSD500 in all three settings by using either ZFNet or VGG16. The reason is as follows. SSD300 resizes images into 300×300, while SSD500 makes the size as 500×500. The region candidates in SSD300 cover a relatively larger area than those in SSD500. Since the lesion region takes a good portion in an image, SSD300 is able to better capture the region, which thus leads to better performance. Furthermore, SSD300+ZFNet is better than SSD300+VGG16 under the benign setting but worse under the malignant setting. This interesting observation can be explained based on the model complexity of ZFNet and VGG16. Specifically, although ZFNet is a small neural network, it can well handle the easier case (i.e., benign), but is a bit underfitting for the harder case (i.e., malignant). In contrast, the larger VGG16 model is good at dealing with malignant lesions, while getting overfitting for the benign ones.

### Classification

For full-images and LROI, AlexNet, ZFNet, and VGG16 perform poorly when learn from scratch, due to the curse-of-dimensionality problem lead to which easy to over-fitting. GoogLeNet uses the inception module as dimension reduction modules to increase the depth and width of network which improved the result than AlexNet, ZFNet, and VGG16 on our dataset. ResNet addresses the degradation problem by introducing a deep residual learning framework, instead of hoping each few stacked layers directly fit a desired underlying mapping, explicitly let these layers fit a residual mapping. Resnet is easy to optimize when the depth increases, and can easily enjoy accuracy gain from greatly increased depth. In Table 2, we can see ResNet gets more accurate than GoogLeNet on our dataset. DenseNet connects each layer to every other layer in a feed-forward fashion to alleviate the vanishing-gradient problem and strengthen feature propagation. DenseNet reduces the number of parameters than traditional convolutional networks in the case of the same number of layers, as there is no need to relearn redundant feature-maps, which has already obtained best result on our dataset.

Potentially, transfer learning could further improve classification performance. In Table 2, in four different scenarios, we observed that all networks transfer learning from the large-scale annotated ImageNet, which produce higher accuracy rate than random initialization, and DenseNet obtain best result.

## Conclusions

In this paper, we have mainly studied the existing state-of-the-art CNN based methods for breast lesion detection and classification in breast ultrasound images. Due to lack of publicly available datasets, in order to analyze and evaluate the methods for CAD in breast ultrasound images, we have collected a new dataset consisting of 579 benign and 464 malignant lesion cases with the corresponding ultrasound breast images, and have them manually annotated by experienced clinicians.

For the detection task, we employ the state-of-the-art CNN based detection methods to locate lesion regions in breast ultrasound images and systematically evaluate them on our newly collected dataset. We establish benchmarks for our newly collected dataset, and our study can potentially benefit other researchers working in the same area. Through comprehensive experiments of detecting the lesion regions, we find that SSD300 achieves the best performance in terms of APR, ARR and F_1_ score.

For the classification task, on our dataset, we systematically analyze the performance of different CNN based classification methods in four scenarios. Our experiments reveal that the deeper network with less parameters obtain better results on our dataset. Transfer learning from the large-scale annotated ImageNet to classify breast lesions significantly improves the performance of different CNN architectures. DenseNet is more suitable for our problems.

Currently, our dataset is based on the ratings obtained from the BI-RADS, In the future, we will build well-annotated dataset which is based biopsy result of every tumor. Also, we will conduct further investigation of the new algorithms to improve the performance.

## Data Availability

The datasets used and/or analysed during the current study available are not publicly available due to the patients’ privacy but are available from the corresponding author on reasonable request.

## Abbreviations

APR: average precision rate
AR: accuracy rate
ARR: average recall rate
BI-RADS: Breast Imaging-Reporting and Data System
CAD: Computer-aided diagnosis
CNN: convolutional neural network
LROI: lesion regions of interest
R-CNN: Region-based convolutional neural networks
ROI: region of interests
RPN: Region Proposal Network
SSD: Single Shot MultiBox Detector
SSD300: SSD with the input size as 300×300
SSD500: SSD with the input size as 500×500
VGG16: Visual Geometry Group
YOLO: You Only Look Once
YOLOv2: YOLO version 2
YOLOv3: YOLO version 3
